# Role of Augmented Reality in Tertiary Care: Qualitative Investigation Using Thematic Analysis

**DOI:** 10.2196/68810

**Published:** 2025-08-14

**Authors:** Jacob Hobbs, Christopher Bull, Caroline Claisse, Mat Elameer, Richard Davison

**Affiliations:** 1 Open Lab, School of Computing, Newcastle University, Floor 1, Urban Sciences Building, Newcastle Helix, Newcastle upon Tyne, NE4 5TG, United Kingdom; 2 Stroke Research Group, Institute for Ageing, Faculty of Medical Sciences Newcastle University, Newcastle upon Tyne, United Kingdom; 3 Games Lab, School of Computing, Newcastle University, Newcastle upon Tyne, United Kingdom

**Keywords:** augmented reality, clinical practice, radiology, surgery, thematic analysis

## Abstract

**Background:**

While augmented reality (AR) as a concept is not new, it is still an emerging technology with a wide range of applications that it could provide value for. In the medical field, AR is becoming ever more prevalent, but while it has been applied to various medical tasks, it is far from commonplace. Radiological imaging has been suggested as one of these applications, and the radiology workflow capacity crisis the United Kingdom’s National Health Service is experiencing is a potential opportunity for technology to alleviate pressure. Understanding clinical stakeholders and current systems is important for identifying design opportunities for developing AR to enhance interactions and gain more from radiological images.

**Objective:**

This study had 3 key aims. First, to build an understanding of the field in the context of AR; second, to understand the stakeholders and workflows surrounding radiological images; and finally, to suggest how AR could integrate within these workflows and current practices in order to provide value.

**Methods:**

We conducted 14 interviews with hospital-based consultants in a range of specialties and then completed a thematic analysis on the transcripts in order to find trends that suggest what value AR could add to radiological imaging, where that value could be added, and who would benefit. We implemented reflexive thematic analysis to develop themes from across the interviews, which were then built on to suggest design implications.

**Results:**

We find that the need for efficiency in image evaluation is present across many roles, regardless of the clinical question, but consultants can be resistant to new technology. Additionally, we find that the current capability of AR technology could be of greater benefit to radiologists as opposed to surgeons or other practitioners. We discuss these findings for the development of AR applications and present 3 design implications that stand as our core contribution.

**Conclusions:**

We conclude with 3 design implications for the application of AR within radiological imaging based on the results of our thematic analysis and frame them within the Human-Computer Interaction and medical fields. The first design implication highlights efficiency and how AR has the potential to allow for quicker comprehension and measurements. Second, we suggest that the capability of AR tools should complement existing techniques and not simply replicate current ability in 3 dimensions. Finally, the integration of AR tools with existing workflows is crucial in the uptake of the technology in order not to negatively disrupt practice.

## Introduction

### Overview

Augmented reality (AR) for clinical use was first mentioned in 1982 [1] and 1992 with a head-mounted display (HMD) [2]. Using AR in a clinical setting is not a new concept, but it is still in relative infancy [3,4] with many suggestions as to the specific applications [4-6]. It is a promising application area of AR with many examples presented [7]. Despite this, it is still an emerging technology, and there is very little uptake of AR in the day-to-day of clinical practice [6]. The motivation of this study is to explore where this emerging technology could provide value to modern medical practices, specifically radiological imaging. The Royal College of Radiologists highlights the urgent workflow capacity crisis in terms of the number of staff not keeping pace with the increasing demand for imaging. Increased strain is therefore placed on existing staff within the National Health Service (NHS), the publicly funded health care system in the United Kingdom [8]. By exploring these problems in the context of AR, we can begin to understand how the technology could fit into the goals or requirements that are present in today’s practice, such as increased efficiency or higher accuracy.

In this study, we conducted an interview study to investigate the current clinical landscape of radiological imaging in modern medicine to better understand the potential roles AR could play and the value it could bring. This was achieved by conducting a set of interviews with consultants in different tertiary care specialisms (highly specialized care) aimed at exploring the current practices and perspectives of professionals who work with radiological images, in the context of using AR technology. As a result, we propose 3 design implications to consider when designing AR systems for clinical use, which stand as our core contribution. Our design implications were informed by experiential accounts and opinions regarding what radiological images are used for, how they are used, and what stakeholders gain from them. We recruited surgeons and radiologists as key stakeholders, and a focus was put on the interactions these stakeholders had with the images used during clinical workflows. This enabled us to examine the contents of the interactions as well as the users’ experiences and opinions on how successful they were in the context of looking for opportunities to design for AR. The current tools used, how the tools are integrated into practice, and opinions on them were also considered.

The aim of these interviews was threefold: to gain an understanding of the field in the context of this technology, to gain an understanding of the stakeholders and workflows surrounding medical images, and to begin to understand the role that AR could play within these workflows. The interviews were semistructured around questions that sought to clarify medical facts, explore the opinions and discrepancies of current practice, while also probing attitudes toward the problems, opportunities, and new technologies that are faced. The interviews have been analyzed using reflexive thematic analysis [9,10] to understand trends and contradictions across the data set. This analysis is intended to understand what value AR could provide in a clinical environment and, therefore, identify application and interaction design opportunities and suggest some design implications. Going forward, this will allow us to begin to identify some of the needs of tertiary care practitioners in the context of this technology. The contribution of this work is the empirical understanding gained through the thematic analysis and the 3 design implications developed based on this analysis. The thematic analysis aims to understand the needs and challenges experienced by hospital-based consultants, and the design implications are developed through and justified by this thematic analysis.

### Background

#### Development of AR

AR superimposes digital objects into the users’ view in real-time using a headset or other device. The aim is to add virtual components to the user’s field of view to provide them with additional information while carrying out a task [5]. Although the term was coined in 1992 [11], the technology has seen a boom in interest in recent years [12]. It was at this early stage in 1992 that AR would be suggested as a tool to aid surgery by Rosenburg [2]. Rosenburg suggested that just as a physical ruler can be used as a tool to aid in drawing a straight line on a piece of paper, AR could be used to guide surgeons’ incisions, and that AR would be better than any physical tool for this task as the virtual components could be partially submerged in the anatomy to strictly follow key lines and boundaries.

Since this time, AR technology has developed with advancements such as viable HMDs, allowing wider and more creative adoption [13]. There is little clinical use of AR, but interest in the technology for use in this space is growing [6]. It has been suggested for image-guided surgery (IGS), as Rosenburg did, but also for tasks such as medical training, clinical psychology, diagnostics, surgical planning, and rehabilitation [5,14,15].

HMDs are the dominant way of using AR, and technological developments have meant that they can display content accurately enough to enable convincing interactions. However, technological and usability issues persist around AR HMDs [16-18], with the effectiveness and accuracy of AR in many clinical tasks difficult to validate and therefore remaining to be proven [19,20]. A key set of issues documented across a variety of AR headsets is the perceptual inaccuracies and issues that can arise. Perceptual issues are an important area of research, as regardless of the domain or individual application, an otherwise perfect AR experience could be made intolerable by physical symptoms as a result of inaccurate perceptual cues. This is particularly true in a medical environment where the accuracy of the tools used can have an implication on a patient’s life [21].

Poor perceptual cues can place stress on a user, resulting in symptoms such as motion sickness, nausea, and visual fatigue. Focal rivalry is a common example of inaccurately represented virtual content, placing unmanageable stress on the users’ vision. Focal rivalry is where the eyes cannot focus on 2 objects at different depths at the same time and therefore have to switch between focusing on the physical object and the virtual, a requirement rarely seen in the natural environment [22] .

The vengeance-accommodation conflict is another common perceptual issue that has been documented to cause physical symptoms. The vengeance-accommodation conflict [23] is caused by the eye’s 2 mechanisms of focusing competing against one another. Most modern HMDs have a fixed focal depth of around 2 m, but as virtual content is moved away from this plane, inaccurate depth cues are created, often out of the bounds of what a user’s eyes can tolerate [24].

#### Gold Standard: AR IGS

AR IGS was one of the first clinical applications AR was suggested for, and is still a key area of interest in medical AR research, and is a clear application of the technology [19]. It can be argued that AR IGS is the gold standard of clinical AR as there is broad agreement that having live guidance for operations would be of significant value to the surgeon, resulting in a higher chance of successful surgery [15,25,26]. The theoretical implementation of IGS is that guides such as 3D virtual representations of anatomy, built from preoperative scans, are overlaid onto the patient in order to allow the surgeon to see anatomy below the surface and more easily identify structures, as well as the boundaries between them. This is intended to speed up procedures, reduce trauma, and reduce recovery time [27].

However, significant issues remain with reaching this goal, which can be broadly divided into technical and usability issues. An important technical issue is registration, the process of aligning virtual components with their physical counterpart. Registration requires enough identifiable points, which can be known as markers, to be present on both the virtual object and the physical anatomy in order to map one to the other, and in a lot of cases, there are not enough. Machine learning algorithms have been used to approach this problem and generate nets of points across both objects, then map them together [28]. Bertolo et al [29] cite registration as a prominent unsolved challenge and state that in the era of “precision surgery,” clinicians will expect error margins to be negligible.

In addition to the technical issues, it is still unclear how best to present virtual content to a surgeon for IGS. Dilley et al [30] suggest that even with perfect registration, surgical performance is reduced when virtual content is overlaid onto the surgical site. Their work suggests that even in a currently fictional environment where perfect registration can be achieved, projecting the images used for guidance beside the patient, unregistered, provides a better outcome.

Determining the best way to present virtual content is one of many usability issues that remain unsolved. Successfully determining what virtual content is best to display to a surgeon can only be useful if the methods the surgeon uses to interact with the content are intuitive, unobtrusive, and effective. The study by Eddie [19] suggests that the visualization and interaction challenges are the biggest challenges facing AR surgical guidance.

AR IGS is likely to provide significant value to surgeons once its value and accuracy can be proven. However, there are multiple issues that all need to be overcome to achieve this. IGS is far from the only application of clinical AR to provide value [31].

#### Modern Clinical Applications

Modern clinical applications of AR can broadly be split into 3 categories: intraoperative (eg, AR IGS discussed above), education and training, and presurgery tasks. The educational and training applications of AR are very broad, ranging from using AR to facilitate the learning of anatomy to safer, more repeatable surgical training [32]. AR has the potential to provide more immersive, repeatable, readily available training and education in the medical field, allowing everyone from medical students to qualified surgeons to take in new knowledge in a new way [4]. In situations where a qualified surgeon is learning a new procedure, AR allows a safer, no-pressure environment for the surgeon to understand how the procedure works and repeatedly practice the intricacies [33].

There are several applications of AR in the presurgery domain, principally, diagnostics and surgical planning. AR for surgical planning allows the surgeon to view preoperative images such as computed tomography (CT) and magnetic resonance imaging (MRI) images, as 3D models of the surgical site before the procedure [15,34]. This is suggested to allow the surgeon to gain a better understanding of the surgical site and relationships between structures, meaning they can plan how a procedure will be approached and be more prepared for potential complications [35].

Douglas et al [36] suggest that using AR could improve diagnostic accuracy and speed up the diagnostic process when viewing cross-sectional images such as CT and MRI. Pelargos et al [37] state that “surgical planning is inherently a 3D task” and that virtual reality and AR technologies could help by improving the understanding of the complex anatomical relationships. These tools have the potential to offer better visualization of areas of interest and therefore improve the understanding and the speed at which decisions can be made [34,38]. Trestioreanu et al [39] argue that AR and virtual reality have the potential to improve radiology health care by improving the cognitive experience, by reducing the cognitive load that a clinician undergoes when viewing 2D slices of 3D anatomy. They go on to suggest that while a few 3D visualization methods currently exist, they do not offer the increased practicality or ergonomics that AR approaches could offer.

As it stands, there is very little AR in day-to-day clinical practice [6,19]. The literature discussed above has directed our work to focus on investigating where AR could be applied in the presurgical domain around radiological images and what value the technology could bring. This is a promising area of research where AR technology could be harnessed effectively. Our work is positioned to direct future research and contributes to the body of literature directing the development of AR applications for radiology, based on expert end user experiences.

## Methods

### Ethical Considerations

This work was granted ethical approval by Newcastle University ethics committee (27432/2022). Participants gave their informed consent to the interviews, and it was made clear that they could withdraw their participation at any time. Ages of participants were captured as ranges and demographic information captured was kept to a minimum to maintain participant privacy. Participants received no compensation for their time.

### Recruitment Process and Participants

For this study, 14 semistructured interviews were conducted with medical professionals from a range of specialties to enable us to determine how practices and perspectives around radiological images vary across specialisms and hospitals. Five of these interviews were with radiologists with various subspecialties, while the remaining 9 were with other consultants in areas such as cardiology, cardiothoracic surgery, general surgery, orthopedic surgery, and clinical oncology. Participant demographic details are summarized in Table 1. All of the participants were male, which is acknowledged and discussed in the Limitations section. Demographic questions were voluntary, and as such, some participants chose not to share some personal information, which is denoted in Table 1 with “—.” The participants worked at 8 different hospitals, 5 of which were in the Northeast and Northwest of England. Two of the remaining were London hospitals, and one on the South coast of England. Initial participants were recruited through the authors’ host university medical school via public staff lists. These participants were then asked to refer other potential participants, especially from other hospitals and regions of the United Kingdom, having a snowballing effect. One of the participants was previously known to the researchers, 2 participants were recruited through mutual acquaintances, and all others were previously unknown to the researchers.

**Table 1.** Participant demographic information.

ID	Age range (years)	Ethnicity	Role	Time in current role
A	45‐54	White British	Consultant interventional cardiologist	19 years
B	45‐54	Mixed White Asian	Consultant cardiologist	11 years
C	45‐54	Indian	Consultant cardiologist	14 years
D	—^a^	—	Consultant oncologist	—
E	55‐64	White British	Cardiac surgeon	20 years
F	55‐64	—	Consultant interventional and diagnostic neuroradiologist	—
G	45‐54	White British	Thoracic surgeon	11 years
H	—	—	Orthopedic surgeon	—
I	45‐54	White	Consultant general surgeon	10 years
J	55‐64	White British	Cardiothoracic surgeon	5 years
K	25‐34	Mixed White Arab	Consultant neuroradiologist	9 months
L	35‐44	White British	Consultant radiologist (nuclear medicine)	10 years
M	35‐44	Indian	Consultant radiologist	4 years
N	35‐44	White British	Consultant cardiothoracic radiologist	4 years

a Not available.

### Interview Process

Semistructured interviews were chosen over fully structured interviews in order to be more open-ended and allow greater flexibility for free conversation. The interviews were all conducted over Microsoft Teams (Microsoft Corp) and lasted between 30 minutes and an hour. Fourteen questions were drawn up based on prior reading in the area, in context with the aims of the interviews. The first objective of the interviews was to act as a means of gaining knowledge of relevant medical specialisms, their current working practices, and collaboration methods across NHS trusts. This way, the authors could build a solid base of knowledge of the field that allowed an appreciation of the context and the identification of nuance in practice. The current practice surrounding radiological images was a key point here. This included establishing how images are used, the tools used to interact with them, how the tools and requirements change between different specialties, and what is gained from the images themselves, that is, what questions they are used to answer. This continued into establishing the current workflows around these images, the communication between stakeholders in reference to imaging, particularly the communication between these hospital-based consultants, how information flows between stakeholders, and what this process looks like from a patient’s perspective.

The clarification of this base knowledge laid the groundwork for more in-depth questions exploring the opinions around these areas: how useful the tools are, how the tools vary, and how personal preference influences both the use of tools and the practice itself. This was then followed by questions about their experience level, their use, and the utility of AR, which were intended to explore the current uptake of this technology and opinions on AR as it exists at the current point. Finally, there were questions about the future of the participant’s specialty and what technologies they saw as having a notable impact.

### Analysis Process

#### Overview

The interviews were recorded and transcribed, providing 14 transcripts that could then be subjected to reflexive thematic analysis. This allowed the authors to establish trends and reveal insights across the whole interview dataset. Thematic analysis is a set of methods for data analysis to develop, analyze, and interpret patterns across a qualitative dataset. Reflexive thematic analysis, developed by Braun and Clarke [40], is an interpretive qualitative approach that encourages critical reflection of the role the researcher plays in the analytic process and their research practice. Braun and Clarke talk about the inherent presence and necessity of biases and how they are integral to reflexive thematic analysis. Reflexivity is integral to this analysis method, “We must question why we think what we think. Bias, prior knowledge and who we are shapes subjectivity” [41]. Thematic analysis is a set of interpretive qualitative analysis methods, and as such, the researchers’ perspectives and biases are used as tools for analysis. It is important to understand these biases when carrying out this form of analysis in order to understand the context used to come to conclusions and how that context influences the conclusions. It is at this point that we, as authors, must consider our positionality.

#### Positionality Statement

We cannot expect the interviews to uncover the full range of opinions and practices within a particular medical specialization, but we aim to include a sufficient range of participants in order to be representative of the area. Where this is not possible, the researchers acknowledge which groups could not be recruited and the effect this may have on the analysis. Authors 1 and 2 (JH and CB), who conducted the analysis, are computer scientists in Open Lab, a Human-Computer Interaction (HCI) laboratory in the School of Computing at Newcastle University, United Kingdom, with experience in digital health, but no formal medical training. Our expertise lies in qualitative methods and designing technologies for specialist user groups. The remaining authors contributed and provided additional context after the analysis was completed. These authors can be considered tech-savvy and protechnological innovation, which will lend the interpretations of the analysis to following this philosophy. Other researchers will bring different perspectives and have different experiences informing their analysis and will, therefore, come to different conclusions.

#### Implementing Reflexive Thematic Analysis

Thematic analysis is an overarching term for a flexible set of methods designed to interrogate qualitative data. This study follows the updated version of reflexive thematic analysis by Braun and Clarke [10], which builds on their original work [40]. It is appropriate for this study as the aim of the analysis was to understand the common themes and contradictions across all 14 interviews in order to provide rich insights across a range of specialties [42]. A reflexive approach was applied to this study to foster an organic coding process and to use researcher subjectivity as a tool. This approach means that “themes cannot exist separately from the researcher—they are generated by the researcher through data engagement” [42] and is a direct result of researcher subjectivity being positively exploited. While thematic analysis is a theoretically flexible set of methods, it is important to understand the theoretical base and assumptions being brought to the analysis [9]. For this study, the authors approached the analysis from a relativist ontological position and used a constructionist epistemology. This means that the authors could explore the meaning from the participants in context and be directed by this, constructing meaning and evidence through the analysis. This is opposed to a more traditional realist postpositivist approach, where it is considered that a single objective truth exists within the data, and it is the researcher’s job to find it [10,43].

As defined by Braun and Clarke [10], an inductive coding process was used in this study. This was to enable the focus to be put on the participants’ experiences and opinions, and as such, allow themes and contradictions between participants to be brought to the surface. As previously mentioned, this inductive process was colored by inherent epistemological and ontological assumptions as “you cannot enter a theoretical vacuum when doing TA” [44]. In a similar vein, a combination of both semantic and latent codes was used throughout the coding process. The semantic codes captured the explicit, surface-level detail that was being communicated while the latent codes grasped the deeper, more implicit points being made. This combination allowed for a thorough and meaningful analysis.

In terms of the analytic process, for reflexive thematic analysis, Braun and Clarke [10] detail 6 phases: familiarization, coding, initial theme generation, developing and reviewing themes, refining, defining and naming themes, and writing up. The familiarization phase was achieved in 2 ways, first, with the lead author conducting the interviews, there was an initial exposure to all of the data in the context it was given. Second, through the transcription process. Automated tools were used for the bulk of the transcription, but the lead author checked each transcript against the interview recording. This ensured that the transcripts were accurate while also contributing to the familiarization phase of the analysis. The coding and theme generation were primarily carried out by the lead author, with the second author offering opinions and challenging decisions after each round. Two full coding rounds were completed, and theme generation was completed over 3 iterations with the second author contributing opinions after the initial coding of 2 transcripts, again after all transcripts had been coded and between iterations of theme development. This contributed to the robustness of the coding and theme generation phases, as it was an opportunity for biases and assumptions to be questioned. The second author contributed to the analysis by reviewing initial codes and themes and probing into the reasoning behind them. This provoked further reflection on the codes and themes throughout the analysis process and meant that assumptions could be challenged, resulting in a deeper meaning being developed. Initial coding resulted in several hundred codes, but upon review, in between and after each coding round, similarities between codes were identified, and clustering codes together allowed for easier interpretation for theming. We then initially grouped codes into 12 broad patterns (eg, multidisciplinary teams [MDTs], personal preference, increased reliance on imaging, and relationships with imaging), which could then be reviewed between authors and the logic or biases challenged. These were then iterated on with the context and theoretical positioning discussed above to develop the themes presented below. Each theme articulates a different aspect of the conversations had while sitting within the context of this work.

## Results

### Overview

The results are presented as the 4 themes developed through the thematic analysis process. These 4 themes are that communication is largely verbal or written, which acknowledges observations around how communication is conducted regarding radiological images and how it is mostly via the radiologists' report and in MDT meetings. Inconsistencies and personal preference in practice encapsulate the extent to which personal preference and other choices change practice. Extended reality (XR) maturity for surgery covers the opinions of current XR technology, AR in particular, and how there is potential for it in certain areas of practice, but there are still significant issues preventing the mass uptake. Finally, increased reliance on imaging is a known issue in radiology, but this theme explores the opinions in this area and the potential ramifications interviewees believe they will experience. These themes are summarized in Table 2.

**Table 2.** Theme table summarizing themes and characteristics.

Theme	Subthemes	Characteristics
Communication is largely verbal or written	MDTs^a^ Written reports An intuition of knowing what questions the next clinician will ask	MDTs and radiologists’ written reports are stored and interacted with via PACS^b^. MDT communication is high-level, aiming to reach decisions quickly.
Inconsistencies and personal preference in practice	Discrepancies in reporting Discrepancies in tools used	Tools, expertise, and practice vary between consultants, departments, and trusts. Given the same tools, slightly different results are likely to be reached.
XR^c^ maturity for surgery	—^d^	Current technological state of the art. The potential impact of AR^e^ technology.
Increased reliance on imaging	Efficiency required Acceptability of new technology	Efficiency is a big concern for everyone, but particularly radiologists. The appeal of new technology to clinicians varies—value must be proven.

a MDT: multidisciplinary team.

b PACS: picture archiving and communications system.

c XR: extended reality.

d Not available.

e AR: augmented reality.

### Communication Is Largely Verbal or Written

Including a variety of hospital-based consultants as participants in this study provided insight into the communication between these 2 parties and how radiological images are used in this process. Two of the important opportunities for communication in terms of radiological images are the radiologist’s written report and the MDTs. The report written by the radiologist with their interpretation of the scan will aim to answer the clinical question that accompanies the scan and will be read by the referrer and any other consultant who has a stake in that patient’s care. Any unrelated incidental findings will also be reported. For straightforward cases, this will be the only communication between the reporter and referrer; more complex cases are likely to be sent to an MDT. These MDTs will have at least one of every specialist relevant to the pathology present, and cases will be discussed as a group with each participant putting forward their views. It was made clear by participants that MDTs were introduced to help make better-informed decisions and to lift the responsibility of decisions from 1 person. Participant L described that these meetings aim to “make a good decision quickly.”

The reports that accompany scans are the key value that radiologists contribute to the point where, for more straightforward cases, a referrer may not look at the images when planning the next step of the patient’s care. Participant A said, “for most relatively simple questions, I would just go by the report.”

The MDTs are the main point where cases are discussed and decisions are made with the full range of expertise. During these meetings, the radiologist will share relevant images and talk through the salient details with the group of specialists so that each can put forward their opinion. The images are not likely to be viewed for an extended period of time here, as MDTs are generally a high-level discussion, and there will be a lot of cases to get through in minimal time.

A trend across the interviews was the notion of knowing what information the next clinician in a patient’s line of care will need in order to do their job, as well as the radiologist sculpting their report and the presentation of information at the MDT toward that. Participant L said, “I do the same MDT every week and have done for 10 years. So we’re a bit more experienced [...] so that we know what they want in those specific circumstances.” They then went on to talk about reporting scans from other hospitals and said, “If you don’t know your referrers you don’t know how they like their reports or whether there are specific things on there they want or things like that. So it’s better to report scans from your hospital for a number of reasons.*”* There is the idea here that knowing, or having an intuition of the next steps of care, will have an impact on how information is portrayed.

Additionally, it is clear that while radiological images are essential to communicating information and making decisions for patients’ care, they play a supporting role and are only the center of attention to the radiologist reporting them. Each step after this, the radiologist distils the information down to the relevant points, chosen based on experience and specifically to answer relevant clinical questions.

### Inconsistencies and Personal Preference in Practice

#### Overview

This theme encapsulates and describes the observed inconsistencies in practice between the range of specialists interviewed, and how much of a role personal preference plays in the details of practice. This is split into 2 subsections: reporting and tools.

#### Reporting

Across the dataset, particularly in the interviews with radiologists, the subjective nature of image analysis was made clear. Most of the radiologists used the term “interpretation.” Participant L said, “And my interpretation of it, if someone else has reported it, I will change if I don’t agree with it” in the context of reviewing cases before an MDT meeting. This subjectivity around the details of reporting presented itself directly through radiologists referencing it and also through radiologists talking about confirming others’ “opinions.” Participant L said, “when I’m allocated to do attending [...] we do get a lot of telephone calls asking for opinions from scans which have been done at other hospitals.” The data suggested that the uncertainty was greatest between departments or between hospitals. Participant N said, “if one of my colleagues has reported it [...] usually I just look at what they’ve said, because I’m always going to agree.” This suggests that within departments, experiences and expertise are shared and therefore create an isolated unit of consistency.

Radiologists also talked about sculpting their reports for those who were going to read them. The radiologist participants made it clear that in many cases, they know how specific consultants like their reports or that they know what questions such a consultant would have, and therefore, they write their report for them. This implies a level of inconsistency around what content should be in a report, and that efficiencies are gained by working with the same people for an extended period of time and getting to know how they work. Additionally, part of medical knowledge comes from the scenarios that individuals have experienced and the results of reactions to those scenarios. Participant N recalled 1 difference between him and a colleague who has recently retired was “He’s coming at it with far more experience and that will colour his opinions of all the things he’s seen and the things I haven’t seen. Likewise in certain areas I’ve trained for more recently than he has so some of the more modern things I might have done a little bit more of.” This experiential part of medical knowledge will likely lead to inconsistency in how scans are reported, as different reporters will bring different knowledge and experiences.

#### Tools

The use of different tools between different departments and trusts was immediately apparent, with personal preference playing a key role.

Picture archiving and communications systems (PACSs) are the systems used in hospitals to store, view, and report radiological images. With many vendors available, it is each NHS trust’s decision which to buy into. While PACS implementations will have a common set of functions, different vendors will have subtly different implementations. This leads to trusts choosing a system that is most appropriate to their specific requirements. As such, interoperability, and in particular, image exchange, between trusts becomes an issue.

The use of third-party tools was a clear example of personal preference throughout the interviews. Third-party tools are a department-level decision, and as such, there was considerable variation in the choices made. Participant J said, “we haven’t bought into any of that market [...] because we think at the moment, if you have a one millimeter or less slice contrast-enhanced scan, with our PACS system, you should be able to reconstruct and see sufficiently.” Conversely, participant N had more than 1 third-party tool available to use and described 1 of the third-party tools they use as “fairly ubiquitous in cardiac MRI.” This demonstrates that there is some consistency regarding the tools that are used within specialties, but across specialties, there are differing views toward the built-in tools available in PACS systems.

Throughout the interviews, there was a lot of conversation about 2D versus 3D methods of viewing cross-sectional radiological images, such as CT images. Most participants saw 2D slices as enough. Participant I said, “You scroll through [the 2D slices] using the mouse wheel and I’m building up a picture going through the images. And I have to say that’s more than enough.” Other participants, both radiologists and surgeons, said similar things. 3D techniques were used in specific scenarios, such as looking at the whole surface of a structure, such as the skull, as noted by participant K. Generally, 3D images were used for specific questions, but participants claimed they did not add very much value beyond that.

However, there were situations where 3D techniques were very valuable. Participant H, a thoracic lung surgeon, used a third-party company to reconstruct cross-sectional scans into highly accurate 3D models to be able to plan their operations better. They commended its value, but due to the cost per case, said it cannot be used for every patient; they said, “the frustration is that we can’t have it for every single patient.” It is clear then that traditional 2D techniques are still dominant, but in certain groups, and in certain scenarios, newer 3D techniques are adding value.

### XR Maturity for Surgery

It was clear throughout the study that radiologists, surgeons, and other consultants have very different relationships with radiological images. This is unsurprising, but the analysis was an opportunity to delineate these relationships and understand the effect that they have on experiences and requirements of current AR or XR systems.

It was evident that radiologists spend a much more extended period looking at images as they have a much broader question to answer. Radiologists will answer the clinical question that accompanies the set of images, but they will also look at the rest of the pictured area and report “incidental findings” if required. These incidental findings are a key point of value that the radiologists add. Surgeons, on the other hand, will be looking to answer very specific questions that may affect or change the operation they are about to conduct. One radiologist participant summarized this difference as “If you have a brain surgeon they’re going to be an expert in looking at things they can operate on [...] But if you showed them something they can’t operate on, like a stroke, they’re not going to recognise it. The radiologist adds value in looking at all the other things on the scan.” An example of this would be a radiologist measuring a key structure pictured in the scan and including this measurement in their report. The surgeon would then take this measurement as information to use when deciding whether or not to operate or when planning how to approach the procedure.

Across all participants, the experience of AR in clinical practice was little to none, and the opinions of current systems were consistent, particularly among the surgeon participants. The view of the current systems on the market indicated that they added very little value, and definitely not enough to overcome the cost of buying into such technology. Participant E, a cardiac surgeon, referred to the systems they had experienced as “perhaps not quite at the gimmick end of the spectrum, moving a little bit away from that, but still there.” There was some inconsistency around opinions as to what role AR could play in the future. Some participants could very much see the potential value in specific areas, while others could not see how AR could improve their current capability or practice in any way. Participant H, an orthopedic surgeon, looked into using a Microsoft HoloLens (Microsoft Corp) to guide the placement of implants, while participant I, a general surgeon, said the presentation of scans as 2D slices is “more than enough” to get the information they need to operate successfully. Participant H acknowledged the potential value of AR for thoracic lung surgery but reinforced the importance of correct registration and how this remains an unsolved issue with the current state of the art.

One of the first things AR was suggested for is IGS, and it is one of the applications that could be most valuable [45]. Most of the surgeons spoken to in this study saw some role for AR to aid surgery as being in the future of their fields. IGS has a very wide scope with many different surgical fields and specific interventions that could benefit from AR, and each will have its own requirements. Robotic surgery is an obvious potential application, as the surgeon is already looking at the operating site through a headset of sorts. Participant J, a robotic thoracic surgeon, when asked about the future said, “there’s got to be more things that can be fed into your vision during your operation” and commending the potential of guidance as a way to reduce risk to patients they said, “there have been times, don’t get me wrong, where I wonder where I am in the chest, and an overlay at that point would be delightful because your fear factor has gone up.” This is a demonstration of where AR could provide tangible value in IGS. It may not be all surgical fields that benefit in this way, though; AR may be introduced in another way. Participant E, a cardiac surgeon, struggled to see how AR could help in their field. Given this constraint, AR may be applied in a different way to add value, such as acting as a head-up display with information like the patient’s vital statistics or a view of the preoperative scans floating above the body to act as guidance in a different way.

### Increased Reliance on Imaging

#### Overview

An increased reliance on imaging is a known issue in radiology [8] within the NHS and has multiple contributing factors, but this is likely to have ramifications throughout the organization. Across the interviews, the requirement for efficiency was ever-present, particularly with the radiologists, as were the acceptability factors that new technologies have to work through in the medical field.

#### Efficiency

Already, there are more scans being taken than can be reported by radiologists, and this is likely to only increase [8] as imaging is an essential part of modern practice [46]. AI for reporting radiological images was brought up regularly in the interviews when talking about the future and efficiency in particular. It was nearly unanimous across all the participants who spoke about it that it would have a big impact on radiology reporting and the number of scans that could be reported in a given time. With an increasing demand being placed on radiologists, the backlog of images to be reported will only grow, increasing waiting times for patients and potentially having negative effects on their care. There was, however, disagreement over exactly how AI would be used. Participant I, a general surgeon, said, “in theory you could replace a radiologist with a computer,” and this was shared among a few others. However, the radiologists saw AI, at least in the foreseeable future, as a tool for radiologists rather than a replacement. Participant K, a neuro-radiologist, said, “having worked in radiology for 6 years and now a year into being a consultant, I think it’s difficult to ever imagine a world in which AI could do everything that a radiologist does,” and participant N, a thoracic radiologist, said, “AI’s got to get pretty good before it’s able to do that because that requires a lot of higher functioning and thought [...]-It’s a tool, and I see it as a tool going forward.”

Similarly to the reporting process, as more imaging is used, MDTs will have to discuss it, and therefore, the process of viewing and manipulating images will have to become more efficient. Radiologists attending an MDT will likely have to review many scans that may have been reported by someone else, quickly, as preparation. Participant N said, “you only get a couple of minutes per case to prep the MDT. Because obviously there’s quite a lot of cases, so I couldn’t realistically re-report every single scan.” It is here that the radiologists check that they agree with how the scan has been reported, particularly in uncertain or complex cases.

#### Acceptability

When looking to the future of medical technology, there were several factors that repeatedly surfaced through the interviews. The first was phrased well by participant N as “technology inertia,” which captures well the resistant nature of the medical field. They went on to say, “I think it’s [the medical field] less open [to new tech], because of the stakes.” This is compounded by other participants saying things such as “people get used to a way of doing things.” This all suggests that even if a new, better technology is available, it takes a significant investment in time and money to implement it in practice. Consultants do not have the time to retrain on new equipment for a very small gain in performance. Current methods are quick through experience and practice and are therefore preferred to retraining. There is a positive attitude toward new and beneficial technology, as evidenced by participant A who said, “I quite like moving with new ideas where possible.” However, this is inconsistent between consultants and not always reflected in the uptake of new technology.

Where there was mention of resistance to new technology, there was often the mention of how age affected this. Participant N said, “to some extent you do rely on younger colleagues coming through to help you innovate, I guess.” This adds to the line of thought that even though new technology may be an improvement, it takes a push to get through the inertia. Just as younger colleagues help the more established to innovate, we must provide a means by which new technology can be effectively demonstrated in order to overcome this inertia.

## Discussion

### Principal Findings

Across all of the themes described in the results section above, there were several linkages. Efficiency came up explicitly and implicitly throughout the interviews, and this is reflected in the themes. There is a persistent reference toward the fact that there are more images taken than can be reported and that this workload is likely to increase [8]. In this vein, there is generally a positive view that new technology has value to provide the medical field, but a contradictory view that current tools, systems, and processes are good enough to obtain the results required and to do the job well. The opportunity here is to understand the clinical requirements and issues being faced and suggest how AR could be used to alleviate this pressure. This section takes the above results and presents 3 design implications as an output, which stand as the core contribution of this work. These design implications, presented at the end of the following subsections, are intended as considerations to be made when investigating the development of AR systems within health care.

After the interviews had taken place and the analysis had been completed, one of the participants was approached to join as a coauthor (author 4). Here, author 4 reviewed the presentation of the clinical side of the analysis and provided further clinical context to the design implications that are presented below.

### Where to Go With the Current Technological Capability

AR has a great deal of value to offer, but it is an emerging technology [47] and has limitations that need to be taken into account when applying the technology. It is important to acknowledge the capabilities of the state-of-the-art AR technology, as well as its limitations. To have this technology deployed in this sector, there must be proof of the value within the limitations. As discussed previously, AR cannot currently reach acceptable margins for IGS. However, over time, as the technology develops, the technological limitations will dissipate, and applications that demand tight margins, such as IGS, will become more feasible. Once AR can be proven to function within the acceptable margins of IGS, there is huge value to be gained [25]. Many of the surgeons interviewed saw the potential value of AR IGS, and the literature supports this [45]. Before this happens, AR still has value to exploit, and it must be determined where the technology can be used to make a difference in its current form. In this section, we suggest radiology as an initial application for integrating AR.

Two key recurring points in our analysis are important here: the desire for efficiency in the workflows around radiological imaging, particularly from radiologists, and the ways in which images are engaged with at each stage of the workflow. Our analysis suggests that there are 2 important points of communication regarding radiological images: the radiologist’s written report and the MDTs. In both the report and the MDTs, the images are, of course, integral, but the time spent on the images after they have been reported can be minimal. This is, in particular, in situations where there is a relatively simple case and the radiologist knows which consultant will be reading the report. They are therefore able to pre-emptively answer the questions the consultant is likely to ask. This matter of minimal time spent looking at images continues to the surgical planning stage. All the surgeons interviewed said that this was a short task where they were looking to answer specific questions that would impact the feasibility of an operation or how an operation would be performed, not a complete reevaluation of the images.

The requirement for efficiency came up repeatedly, particularly from the radiologists’ point of view, and this is consistent in the literature [8]. As discussed previously, the reporting of scans is going to have to become more efficient as the number of scans taken already exceeds the number of scans that can be reported. This extends to the radiologists’ preparation for MDTs, where each case must be reviewed by the radiologist attending the MDT in advance.

In response to these points, we suggest radiology as a starting point for integrating AR into health care, as we believe that the inherent interaction benefits of AR are well placed to be exploited when viewing 3D images. This could give radiologists a better appreciation of the anatomy in a shorter period of time and help them understand relationships between key structures. It may also be used here to take more accurate, quicker measurements of key structures that could help surgeons be better prepared for interventions. This could be of benefit in terms of efficiency.

In addition to this, radiologists spend a significant amount of time with the scans for each case, much more than any other clinician at any other stage in the workflow. This means that the value of using AR can be maximized, and limitations such as the cost of equipment and the learning curve of using it are limited.

Establishing AR in radiology could then allow some usability, procedural, and technological issues to be researched further as part of this deployment of AR. This could then prepare the technology for future deployment in scenarios where there are currently other limitations. Using this as an opportunity to research AR usability in health care, while adding value to the clinical workflow, would be invaluable, as usability issues are as much of a limiting factor to the implementation of AR as technological issues.

This leads to our first design implication: acknowledging AR technology’s limitations and the benefits it can provide, namely the interaction potential, AR should be exploited to help increase the efficiency of radiologists reporting scans. This should be followed by clinical evaluations proving the efficacy of the technology, which may then encourage research into expanding the technology into other disciplines as the technological limitations are mitigated with continued development.

Acknowledging the technology’s limitations and working with its advantages will allow value to be added to processes almost immediately. We argue that radiologists are well placed to exploit value from the interactions that existing AR technology affords, likely resulting in increased efficiency; whether that is the whole reporting process or a subset of tasks such as taking measurements.

### 3D Views Complement 2D Views

Throughout the interviews, 2D versus 3D viewing methods of cross-sectional scans, such as CT and MRI, were a key discussion point. The overwhelming majority were of the opinion that 2D slices of scans in 3 planes were more than enough to gain the information that they required. Some went on further to say that 3D methods lose something over 2D because it is more difficult to look at the internal structures. This was contested in a minority of situations where 3D methods had various specific application areas, such as looking at the surface of the skull and reconstructing lung scans for planning resections. The general consensus was that 3D reconstructions are useful for very specific tasks but add little beyond that.

This suggests, and is intuitive, that the main issue with 3D methods for the participants is the inability to see the same internal structure information that is shown with traditional 2D slices. There was no direct issue with 3D forms; rather, the current 3D viewing methods do not add any value. The opportunity here is to use AR to provide the same information that traditional 2D slices provide while adding value with the third axis. This may enable the radiologist to appreciate the information of the internal structures in the context of the full 3D form in a more intuitive manner. This could also enhance communication and allow a greater shared understanding.

There are examples of using AR in such ways [48], but this interaction has yet to be proven. In order to be accepted by radiologists, the scans shown in 3D in AR must show at least as much information as 2D slices while providing additional value in some other way, such as an enhanced interaction. This value is likely to be in the interaction, as viewing 3D anatomy in 2D images is less intuitive than viewing it in 3D, where further context and relationships may be more visible. The point here is to demonstrate the additional value that AR can provide. This may be difficult, as our analysis suggested that the medical field is quite resistant to change and new technologies. But if it can be demonstrated well and the value translates into better appreciation of structures, quicker turnaround time, or higher throughput, AR will likely become commonplace in radiology offices.

There is clearly big potential in AR IGS, our analysis and the literature [25] show this, but both also show that it is one of the most challenging areas of research. As discussed previously, there are multiple technological issues and usability issues that need to be resolved to unlock this value that are well documented in the literature, with some suggesting that usability considerations of AR are among the most significant potential barriers to the technology’s success [19]. A creative, out-of-the-box approach to these usability problems could allow the successful implementation of AR in health care and, therefore, be a source of great value, allowing the benefits that the technology affords to be exploited in a much wider number of scenarios.

Here we argue for the creative implementation of AR, playing to the strengths of the technology and not simply recreating existing capability in a new medium.

As with the example above, using 3D viewing methods has limited use in current practice, and 2D views are dominant. But given the third axis and immersiveness that AR provides, do 3D views provide something that is difficult in 2 dimensions? For example, better appreciation of complex relationships between structures. Or are 3D images easier to interact with, providing an easier or more accurate way to take measurements of structures of interest?

Designers must be explicit about why AR is appropriate for the application and what value it provides while using creative practices in order to realize the full potential of AR. This is the second design implication we suggest: creativity must be used in the implementation of AR; simply recreating existing capability in a new medium should be avoided, and the strengths of AR should be played to in order to add value to the clinical scenario while maintaining prior ability. In the context of 2D versus 3D images, this could mean that the information provided with 2D slices is still available, while also providing additional contextual information with the third dimension.

### What Does an “Augmented-Reality-First” World Look Like?

Our analysis suggests that there would be limited value in applying current AR technology individually to surgical planning or for use in MDTs, as current imaging techniques give consultants adequate information to make the decisions necessary in these situations. Furthermore, the images themselves are not used for a very long period for these tasks, and as such, the value gained from viewing the images in AR would have to be great in order to be worth the cost of the equipment and the time taken to put on, boot up, and engage with an AR headset. This is in addition to the initial strain of rewriting procedures around the new technology and the learning curve of engaging with the new medium.

This can be held true for today’s “desktop-first” world, where keyboard and mouse are universally dominant. But looking down the road as AR technology develops and its presence increases in daily life, this is likely to change. In this scenario, where an AR headset could be an extension to a desktop environment, the previous limitations (of cost, learning curve, and clinical practice adjustment) are negated, and the cost-benefit ratio of AR in these situations becomes more amenable.

In this “AR-first” world, the use of an AR headset is as embedded in practice as the use of a normal monitor. There is likely to be a set of tasks that clinicians complete that could be improved in some way with AR. Reporting scans, MDTs, and surgical planning could be 3 examples. For these tasks, the headsets would be ready to run alongside, or instead of, the main desktop environment, and as such, the setup and engagement obstacles are averted. AR would be seamlessly integrated into practices, enabling the benefits to be exploited and made the most of. It is this concept of integration that came up repeatedly in different forms throughout the analysis, for example, learning curve, rewriting processes, resistance to new technologies, and efficiency.

Thinking about speculative scenarios such as this, where certain obstacles are put to one side, allows us to highlight other potentially more nuanced concerns and opportunities that should be considered when designing AR applications for this space. It also allows speculative consideration of the breadth of value the technology could bring in isolation, without being overshadowed by current technological or procedural limitations.

The integration of any new technology into clinical practice can be as significant a hurdle as developing the technology itself, with many concerns residing under the umbrella of “integration”; things such as cost, learning curve, and the rewriting of procedures. However, for the AR, what could be gained if the technology is successfully integrated in the right places? Our analysis suggests that AR brings value in its versatility. It will never be at its best if only used for 1 task. The highest value will be attained when many AR-enhanced tasks are considered. If an AR headset were integrated into practice and ready to deploy for several smaller tasks (such as reporting scans, discussing images in MDTs, and viewing images for surgical planning), much more value would likely be gained relative to implementing just one of those examples.

The first hurdle of successfully integrating AR into 1 point in a workflow and proving value for this one task will likely result in the technology cascading into surrounding tasks, slowly reaching toward maximizing the cost-benefit ratio.

Our analysis suggests 2 main factors would have to be proven to enable an “AR-first” environment. First, is the cost-benefit ratio of the technology. It must be demonstrated that the number of tasks AR could be used for and the benefit that it provides in each of them is worth the cost of buying into the technology. Second, the technology must be integrated into practices well enough to the point where putting on and starting up the headset is not an obstruction to the work being done. This will be a significant challenge as it requires the rewriting of some practices and, therefore, a learning curve when using the systems for the first time. It also requires more targeted human-centered HCI research as opposed to a sole focus on the development of AR technology. Targeted HCI research could map this space more effectively, solving some usability issues and laying the groundwork for more advanced AR technology to stand on.

This leads to the final design implication: AR brings value through its versatility. To obtain the most out of this versatility, it must be considered how AR tools integrate with existing workflows and how they will be used in order to create a seamless transition toward wider uptake of the technology. The technology should be integrated in such a way that negative disruption to existing workflows is avoided and maximum value can be gained from multiple workflows.

### Future Work

These design implications aim to help direct and inform future research, while also aiding in decision-making when developing AR applications in this space. Future work will develop these design implications further and test their feasibility by developing a case study application. This case study will conduct further user research and then incorporate the outcomes of this with these design considerations into a prototype. This prototype will then be evaluated by users against the design implications.

This work could also be expanded by focusing on medical education and training. We chose to focus on the clinical radiological applications of AR for this study to contain the scope and focus the design implications. However, participants mentioned educational and training applications, and there is literature supporting their development. Future work could be done to expand or develop these design implications in this space.

### Limitations

Our qualitative analysis aims to provide a representative insight into the views and opinions of hospital-based consultants in the United Kingdom along with their views on AR and the role it could play in radiological imaging. However, we must acknowledge the limitations of both the methodology and the dataset.

Our participants were hospital-based consultants, largely from the North East of England, with a few from the North West and South. We successfully recruited a range of participants with a range of specialisms to provide a variety of views and differing contexts, which adds strength and breadth to this work. However, a potential shortcoming of this participant pool was our ability to only recruit men. Where possible, we took appropriate steps to try and recruit women, but in part due to this being a very male-dominated field [49], we were unable to. This will restrict the gender diversity of the perspectives presented, but it reflects the wider demographic trend in some specialties. Future work should aim for a more diverse participant pool.

Our study was limited to the United Kingdom, which we acknowledge may limit the generalizability to wider audiences. However, this limitation is commensurate with the scope of this work.

We also focused heavily on AR for radiological imaging with little mention of AR for education or training. That is not to say that AR should not be applied to these areas, and it was brought up by participants in multiple interviews. However, for this study, we chose to focus on AR for radiological imaging in order to focus on the design implications.

### Conclusions

In this paper, we have presented the results of a thematic analysis of interviews with hospital-based consultants in order to investigate the role AR could play in radiological imaging. We contribute 3 design implications for AR systems within radiological imaging workflows based on the results of our qualitative analysis and frame them in the context of the HCI and medical fields.

The first design implication outlines the desire for efficiency. AR has the potential to provide enhanced interactions, which could allow for a better appreciation of the anatomy and quicker measurements. Radiologists are well placed to exploit this value as a tool to improve efficiency because being able to view and interpret images quickly would allow them to have a higher throughput. Second, we suggest that AR tools need to be built in such a way that no capability available with existing 2D desktop workflows is lost either by using AR to complement existing 2D workflows or by integrating the 2D capability into AR. Finally, AR tools need to integrate and be interoperable with existing radiology systems to minimize disruption to existing workflows, for example, ensuring compatibility with PACS. The value of AR could be exploited across health care organizations if the technology is integrated well, and we speculate on the impact of what an “AR-first” world may look like and how clinical practices may change were this to happen.

This work also adds to the body of literature acknowledging active surgeons’ opinions toward the potential value of AR IGS and motivates areas of future research into AR’s place around radiological images.

## Abbreviations

AR: augmented reality
CT: computed tomography
HCI: Human-Computer Interaction
HMD: head-mounted display
IGS: image-guided surgery
MDT: multidisciplinary team
MRI: magnetic resonance imaging
NHS: National Health Service
PACS: picture archiving and communications system
XR: extended reality

## References

[1] Kelly PJ, Alker GJ, Goerss S (1982). Computer-assisted stereotactic laser microsurgery for the treatment of intracranial neoplasms. Neurosurgery.

[2] Rosenberg L (1992). The use of virtual fixtures as perceptual overlays to enhance operator performance in remote environments. https://apps.dtic.mil/sti/html/tr/ADA292450/.

[3] Feng Z, Been LDH, Mark B Trends in augmented reality tracking, interaction and display: a review of ten years of ISMAR.

[4] Dhar P, Rocks T, Samarasinghe RM, Stephenson G, Smith C (2021). Augmented reality in medical education: students’ experiences and learning outcomes. Med Educ Online.

[5] Vinci C, Brandon KO, Kleinjan M, Brandon TH (2020). The clinical potential of augmented reality. Clin Psychol: Sci Pract.

[6] Eckert M, Volmerg JS, Friedrich CM (2019). Augmented reality in medicine: systematic and bibliographic review. JMIR mHealth uHealth.

[7] Barcali E, Iadanza E, Manetti L, Francia P, Nardi C, Bocchi L (2022). Augmented reality in surgery: a scoping review. Appl Sci (Basel).

[8] (2023). Clinical oncology census reports. Royal College of Radiologists.

[9] Braun V, Clarke V (2019). Reflecting on reflexive thematic analysis. Qual Res Sport, Exercise and Health.

[10] Braun V, Clarke V (2021). Thematic Analysis: A Practical Guide.

[11] Caudell TP, Mizell DW Augmented reality: an application of heads-up display technology to manual manufacturing processes.

[12] McCarthy CJ, Uppot RN (2019). Advances in virtual and augmented reality—exploring the role in health-care education. J Radiol Nurs.

[13] Domingues GC, Vieira V, Yoshida L What if video see-through in hmds changes how accurately we perform tasks?.

[14] Yeung AWK, Tosevska A, Klager E (2021). Virtual and augmented reality applications in medicine: analysis of the scientific literature. J Med Internet Res.

[15] Sun P, Zhao Y, Men J (2023). Application of virtual and augmented reality technology in hip surgery: systematic review. J Med Internet Res.

[16] Westermeier F, Brübach L, Wienrich C, Latoschik ME A virtualized augmented reality simulation for exploring perceptual incongruencies.

[17] Lystbæk MN, Pfeuffer K, Langlotz T, Grønbæk JES, Gellersen H Spatial gaze markers: supporting effective task switching in augmented reality.

[18] Prinz LM, Mathew T Support lines and grids for depth ordering in indoor augmented reality using optical see-through head-mounted displays.

[19] Eddie PJ (2021). Digital Surgery.

[20] Malhotra S, Halabi O, Dakua SP, Padhan J, Paul S, Palliyali W (2023). Augmented reality in surgical navigation: a review of evaluation and validation metrics. Appl Sci (Basel).

[21] Ferrari V, Cattari N, Fontana U, Cutolo F (2022). Parallax free registration for augmented reality optical see-through displays in the peripersonal space. IEEE Trans Vis Comput Graph.

[22] Gabbard JL, Mehra DG, Swan JE (2019). Effects of AR display context switching and focal distance switching on human performance. IEEE Trans Vis Comput Graph.

[23] Erkelens IM, MacKenzie KJ (2020). 19‐2: vergence‐accommodation conflicts in augmented reality: impacts on perceived image quality. Symp Digest of Tech Papers.

[24] Condino S, Carbone M, Piazza R, Ferrari M, Ferrari V (2020). Perceptual limits of optical see-through visors for augmented reality guidance of manual tasks. IEEE Trans Biomed Eng.

[25] Birlo M, Edwards PJE, Clarkson M, Stoyanov D (2022). Utility of optical see-through head mounted displays in augmented reality-assisted surgery: a systematic review. Med Image Anal.

[26] Eves J, Sudarsanam A, Shalhoub J, Amiras D (2022). Augmented reality in vascular and endovascular surgery: scoping review. JMIR Serious Games.

[27] Fuchs H, State A, Pisano ED, Goos G, Hartmanis J, Höhne KH, Kikinis R (1996). Visualization in Biomedical Computing.

[28] Brunet JN, Mendizabal A, Petit A, Golse N, Vibert E, Cotin S, Shen D, Liu T, Peters TM, Staib LH, Essert C, Zhou S, Yap PT, Khan A (2019). Medical Image Computing and Computer Assisted Intervention – MICCAI 2019, Lecture Notes in Computer Science.

[29] Bertolo R, Hung A, Porpiglia F, Bove P, Schleicher M, Dasgupta P (2020). Systematic review of augmented reality in urological interventions: the evidences of an impact on surgical outcomes are yet to come. World J Urol.

[30] Dilley JWR, Hughes-Hallett A, Pratt PJ (2019). Perfect registration leads to imperfect performance: a randomized trial of multimodal intraoperative image guidance. Ann Surg.

[31] Palumbo A (2022). Microsoft HoloLens 2 in medical and healthcare context: state of the art and future prospects. Sensors (Basel).

[32] Parsons D, MacCallum K (2021). Current perspectives on augmented reality in medical education: applications, affordances and limitations. Adv Med Educ Pract.

[33] Rangarajan K, Davis H, Pucher PH (2020). Systematic review of virtual haptics in surgical simulation: a valid educational tool?. J Surg Educ.

[34] Dallas-Orr D, Penev Y, Schultz R, Courtier J (2020). Comparing computed tomography-derived augmented reality holograms to a standard picture archiving and communication systems viewer for presurgical planning: feasibility study. JMIR Perioper Med.

[35] Izard SG, Méndez JAJ, Palomera PR, García-Peñalvo FJ (2019). Applications of virtual and augmented reality in biomedical imaging. J Med Syst.

[36] Douglas DB, Wilke CA, Gibson JD, Boone JM, Wintermark M (2017). Augmented reality: advances in diagnostic imaging. MTI.

[37] Pelargos PE, Nagasawa DT, Lagman C (2017). Utilizing virtual and augmented reality for educational and clinical enhancements in neurosurgery. J Clin Neurosci.

[38] Elsayed M, Kadom N, Ghobadi C (2020). Virtual and augmented reality: potential applications in radiology. Acta Radiol.

[39] Trestioreanu L, Glauner P, Meira JA, Gindt M, State R, Glauner P, Plugmann P (2020). Innovative Tech- Nologies for Market Leadership: Investing in the Future, Future of Business and Finance.

[40] Braun V, Clarke V (2006). Using thematic analysis in psychology. Qual Res Psychol.

[41] Fine M (2021). Disruptive Voices: The Possibilities of Feminist Research.

[42] Braun V, Clarke V (2021). Can I use TA? Should I use TA? Should I not use TA? Comparing reflexive thematic analysis and other pattern‐based qualitative analytic approaches. Couns and Psychother Res.

[43] Creswell JW, Poth CN (2016). Qualitative Inquiry and Research Design: Choosing Among Five Approaches.

[44] Braun V, Clarke V (2021). One size fits all? What counts as quality practice in (reflexive) thematic analysis?. Qual Res Psychol.

[45] Glas HH, Kraeima J, van Ooijen PMA, Spijkervet FKL, Yu L, Witjes MJH (2021). Augmented reality visualization for image-guided surgery: a validation study using a three-dimensional printed phantom. J Oral Maxillofac Surg.

[46] Łoginoff J, Augustynowicz K, Świąder K (2023). Advancements in radiology and diagnostic imaging. J Educ Health Sport.

[47] Xiong J, Hsiang EL, He Z, Zhan T, Wu ST (2021). Augmented reality and virtual reality displays: emerging technologies and future perspectives. Light Sci Appl.

[48] (2024). apoQlar.

[49] Barnes KL, McGuire L, Dunivan G, Sussman AL, Kee RM (2019). Gender bias experiences of female surgical trainees. J Surg Educ.

